# *Podocarpaceae* and *Cupressaceae*: A tale of two conifers and ancient adhesives production in South Africa

**DOI:** 10.1371/journal.pone.0306402

**Published:** 2024-11-13

**Authors:** Rivka Chasan, Margaret-Ashley Veall, Liliana Iwona Baron, Alessandro Aleo, Paul R. B. Kozowyk, Geeske H. J. Langejans

**Affiliations:** 1 Faculty of Mechanical Engineering, Delft University of Technology, Delft, The Netherlands; 2 Department of Life Sciences and Systems Biology, University of Turin, Turin, Italy; 3 Department of Canadian Heritage, Canadian Conservation Institute, Ottawa, Canada; 4 Department of Chemical Engineering, Delft University of Technology, Delft, The Netherlands; 5 Palaeo-Research Institute, University of Johannesburg, Johannesburg, Gauteng, South Africa; Fisheries and Oceans Canada, CANADA

## Abstract

Research on ancient adhesives from the South African Stone Age is expanding, driven by excellent preservation conditions of adhesives and the potential to address diverse archaeological questions. These adhesives are primarily characterized through microscopic and chemical analysis. Despite geographic variability, a consistently identified component is *Podocarpus* resin or tar. We challenge these identifications, considering another *Podocarpaceae* genus, *Afrocarpus*, and the *Cupressaceae* genus *Widdringtonia*. Gas Chromatography-Mass Spectrometry was employed to analyze molecular signatures of modern wood, tar, resin, and seed cones from these genera. The results form an extensive reference database and reveal challenges in distinguishing these genera based on the diterpenoid signature. While *Podocarpus* is frequently cited, we advocate for a broader classification as *Podocarpaceae* when phenolic diterpenoids are found in high abundances and pimaranes and abietanes in lower abundances, and *Widdringtonia* when the opposite is true. The study differentiates materials used in adhesive production, including leaves and wood, highlighting the significance of α,ω-dicarboxylic acids, hydroxy acids, *n*-alkanes, and alcohols. Tars produced from leaves are characterized by odd-numbered *n*-alkanes, while tars produced from twigs and branches are characterized by long-chain α,ω-dicarboxylic acids, hydroxy acids, and alcohols. Because the differences between these adhesives in terms of raw material procurement and production are great, a more nuanced and cautious approach that acknowledges the challenges in differentiating tree species on a molecular level and considers archaeological and environmental context is required.

## 1. Introduction

Research on South African Middle and Later Stone Age adhesives is a growing field due to the excellent preservation of lipids the deposition conditions provide and the array of archaeological questions that can be addressed [1–3]. When identified, the adhesives are found adhering most commonly to lithics but also to ceramics and bone tools and as free lumps [1, 4–9]. Adhesive research focuses on the microscopic and chemical characterization of the adhesive components. Ingredients that are commonly found include conifer resin and tar, *Euphorbia* latex, plant and animal derived wax, animal fat, and a variety of mineral additives. Although the archaeological adhesive finds are found across South Africa, covering many different biomes, the primary component identified is surprisingly monotone. *Podocarpus*, a genus of conifers, is most frequently referenced in case studies spanning both the Middle and Later Stone Ages [4–8].

*Podocarpus* is endemic to South Africa [10], and it is attested in the archaeological record by burnt wood remains dating as far back as 75,000 years ago [11–13]. Pollen records show that while abundance varied regionally and diachronically, *Podocarpus* was prolific throughout the Stone Age, with clear forests [14–17], and it is perhaps because of this that *Podocarpus* is at the focus of archaeological discourse. Used today almost exclusively for its timber [18], *Podocarpus* can also be transformed into an adhesive. Experimental studies suggest that the tar was produced from the leaves [3], which contain resin channels [19], rather than the bark, which does not contain resin channels [20–22]. When prepared with certain methods, this tar is significantly stronger than adhesives produced from other local plants [3]. The use of *Podocarpus* is reinforced by the molecular analysis of ancient adhesives [4–8]. Here phenolic diterpenoids, specifically ferruginol, sempervirol, totarol, and their derivatives, are used to identify *Podocarpus* [23].

We question the past identification of archaeological adhesives produced from *Podocarpus* for several reasons. First, *Podocarpus* is part of the *Podocarpaceae* family, which contains another genus endemic to South Africa–*Afrocarpus*. While initially clustered together, these genera are distinct [24] and have different leaf anatomies and reproductive systems [25–27]. *Afrocarpus* must be considered as a potential adhesive source, and it is unclear if the two genera can be chemically distinguished. Second, the chemical signature of *Podocarpaceae* is similar to some members of the *Cupressaceae* family [23, 28], represented in South Africa by the genus *Widdringtonia* [10]. Charred wood remains at some archaeological sites [11] support the presence of this plant; however, *Widdringtonia* has been rejected as a potential adhesive source despite the bark’s high resin content because the resin was considered qualitatively inferior [3]. None the less, this does not imply that the resin was not exploited, as the adhesives properties can be improved with the use of additives or with differential treatment [9, 29–31], and qualitatively inferior resins are known to have been used in some instances in favor of tars [32]. Third, besides the leaves there are other parts of the *Podocarpaceae* plants that contain diterpenoids and/or resin, including the wood [28, 33] and the female seed cones [26, 34]. Wood is a known raw material used in ancient adhesive production. In species with resin channels, the resin can be extracted manually, but the wood can also be transformed into tar [35, 36]. In addition, ethnographic research shows that some populations use fruits containing latex to produce adhesives [37].

To address these discrepancies, this study applies Gas Chromatography-Mass-Spectrometry (GC-MS) to characterize the molecular signature of resin from different conifers native to South Africa, including those from the *Podocarpus*, *Afrocarpus*, and *Widdringtonia* genera. We synthesize the results of two separate case studies conducted between 2016–2023 that applied different instrumentation and analytical conditions. Unmodified resin, wood, and seed cones and tar made from leaves and branches were studied to test for molecular variation based on genus and plant part. We propose that these results can be used to reevaluate our understanding of the archaeological record and adhesive production in the South African Stone Age, allowing for a more nuanced identification of what tree species and parts of the trees people exploited.

## 2. Material and methods

Material was tested from two families–*Podocarpaceae* and *Cupressaceae* (Table 1). Within the *Podocarpaceae* family, there are two analyzed genera–*Afrocarpus* and *Podocarpus*. Specimens from four species of *Podocarpaceae* were analyzed.–*A*. *falcatus*, *P*. *elongatus*, *P*. *henkelii*, and *P*. *latifolius*. *Cupressaceae* in South Africa is represented by *Widdringtonia* [27]. Specimens of two species were analyzed: *W*. *cedarbergensis* and *W*. *nodiflora*. The distribution of these plants is variable with commonly *Afrocarpus* and *Podocarpus* populating the temperate coastal regions and *Widdringtonia* populating mountainous regions [38, 39]. Specimens were collected from botanic gardens in the Netherlands, South Africa, and the United Kingdom.

**Table 1:** Overview of the type and number of samples collected from *Podocarpaceae* and *Cupressaceae* species.

Species	Wood	Tar from branches	Tar from leaves	Resin	Seed cones	Total no. samples per species
***A*. *falcatus***	2	3	1	0	0	**6**
***P*. *elongatus***	1	2	0	0	1	**4**
***P*. *henkelii***	2	3	1	0	0	**6**
***P*. *latifolius***	1	1	1	0	1	**4**
***W*. *cedarbergensis***	1	2	0	0	0	**3**
***W*. *nodiflora***	1	1	0	1	0	**3**
**Total no. samples per material**	**8**	**12**	**3**	**1**	**2**	**26**

**Fig 1:**
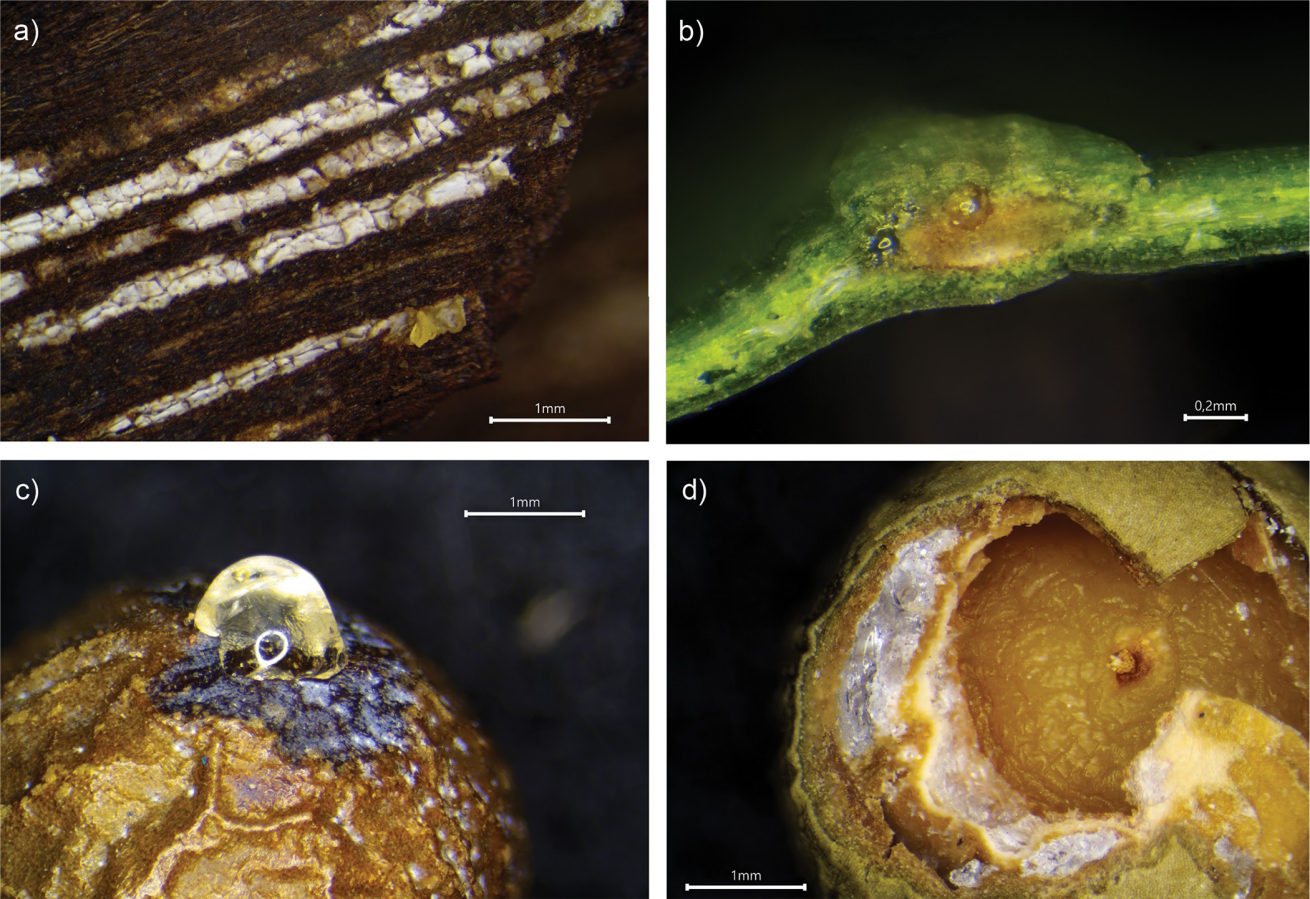
Macroscopic photo of a) Resin exuding from *W*. *nodiflora* bark; b) Resin channels in a *P*. *henkelii* leaf; c) Resin exuding from a *P*. *elongatus* seed when pressure is applied; d) Resin pockets in an *P*. *elongatus* seed.

The wood of *Widdringtonia* is resinous (Fig 1A), and while the wood of *Podocarpaceae* trees does not actively exude resin and is lacking resin channels, it is known to contain terpenoids [28, 40, 41]. The leaves of the *Afrocarpus* and *Podocarpus* trees also contain multiple resin channels (Fig 1B) [19]. Accordingly, tar was produced from small bark bearing branches of all collected species and the leaves of *Afrocarpus* and *Podocarpus* samples (Table 1). The specific tar production methods are described in the S1 File. In addition, unaltered samples of wood and seed cones, the latter of which contain resin pockets (Fig 1C and 1D) [34, 42], were collected as well as one pure resin sample (Table 1).

Sub-samples of the tars and other plant material underwent lipid residue analysis. Three primary extraction and analysis protocols were used (see S1 File for additional information). At TU Delft, lipids were extracted using dichloromethane. At the University of Pisa and the University of Oxford, samples (tars produced from wood) were saponified with a hydroalcoholic solution of potassium hydroxide and divided into neutral and acid fractions [43, 44]. Because of the saponification process, these samples are expected to have different compositions to other pre-treatment procedures, including long chain dicarboxylic acids and hydroxy acids that form during the alkaline hydrolysis and transesterification of suberin. Additionally, at the University of Oxford, samples were extracted utilizing hexane, dichloromethane, and methanol. All samples were silylated using bis(trimethysilyl)trifluoroacetamide (with 1% trimethylchlorosilane). Following GC-MS analysis, the resulting chromatograms were interpreted using the National Institute of Standards and Technology (NIST) library and a prepared AMDIS library. Reference mass spectra for all discussed diterpenoids are provided in the S2 File, and the general fragmentation pattern is presented here (Table 2).

**Table 2:** List of diterpenoids identified in the *Afrocarpus*, *Podocarpus*, and *Widdringtonia* samples and their fragmentation patterns.

Molecule	MW	*M/Z* values of characteristic fragment ions (% abundance)
**2,3-Dehydroferruginol, TMS**	356	73 (100), 274 (88), 356 (83), 341 (33), 271 (31), 299 (28), 285 (27), 357 (27), 231 (26), 272 (24)
**Abietatriene**	270	255 (100), 270 (34), 173 (32), 159 (32), 43 (24), 256 (20), 185 (16), 69 (14), 58 (10), 271 (7)
**Abietic acid, TMS**	374	256 (100), 73 (42), 241 (35), 257 (25), 374 (19), 32 (18), 213 (16), 185 (16), 105 (13), 359 (13)
**Carboxynortotarol, diTMS**	460	327 (100), 445 (66), 285 (30), 355 (22), 460 (20), 73 (18), 247 (16), 313 (14)
**Communic acid, TMS**	374	73 (100), 81 (35), 79 (29), 119 (29), 105 (26), 91 (23), 93 (22), 175 (20), 134 (19) 75 (19)
**Dehydroabietic acid, TMS**	372	239 (100), 73 (54), 240 (21), 43 (16), 171 (14), 75 (12), 173 (12), 357 (12), 41 (12), 255 (11)
**Ferruginol, TMS**	358	73 (100), 358 (99), 343 (76), 359 (31), 247 (30), 261 (29), 344 (23), 69 (23), 273 (21), 259 (20)
**Hydroxyferruginol, diTMS**	446	446 (100), 341 (87), 73 (49), 447 (47), 342 (29), 431 (21), 75 (21), 259 (19), 448 (18), 299 (18)
**Hydroxytotarol, diTMS**	446	341 (100), 431 (44), 73 (44), 342 (33), 343 (26), 357 (24), 446 (20), 372 (20), 432 (16), 261 (14)
**Isopimaric acid, TMS**	374	73 (100), 256 (73), 241 (61), 257 (30), 41 (27), 55 (27), 75 (27), 81 (25), 109 (21), 43 (19)
**Kaur-16-ene**	272	257 (100), 272 (77), 229 (54), 123 (47), 125 (47), 69 (45), 105 (44), 147 (44), 81 (38)
**Pimaric acid, TMS**	374	73 (100), 121 (63), 120 (30), 257 (24), 75 (21), 41 (18), 55 (18), 81 (18), 91 (17), 79 (15)
**Sandaracopimaric acid, TMS**	374	121 (100), 73 (94), 120 (45), 359 (37), 257 (34), 91 (30), 241 (26), 81 (25), 105 (23), 75 (22)
**Sempervirol, TMS**	358	343 (100), 344 (32), 358 (29), 73 (18), 247 (14), 273 (12), 359 (9), 261 (9), 345 (8), 259 (6)
**Sugiol, TMS**	386	357 (100), 372 (59), 73 (42), 358 (29), 373 (18), 289 (14), 275 (14), 315 (11), 287 (10), 359 (8)
**Totarol, TMS**	358	343 (100), 247 (43), 358 (38), 34 (31), 73 (26), 273 (23), 359 (13), 261 (13), 248 (10), 274 (9)
**Unknown ketone (m/z 261), TMS**	386	261 (100), 372 (46), 275 (27), 262 (23), 373 (14), 301 (11), 357 (9), 217 (8), 276 (7), 73 (7)
**Unknown ketone (m/z 275), TMS**	386	275 (100), 261 (83), 372 (37), 276 (24), 262 (22), 73 (17), 357 (15), 301 (15), 287 (10), 259 (10)
**Unknown pimarane (m/z 241), TMS**	374	241 (100), 73 (27), 359 (22), 256 (15), 374 (12), 173 (11)

## 3. Results

GC-MS was used to identify the following molecule types: fatty acids, alcohols, *n*-alkanes, α,ω-dicarboxylic acids, hydroxy acids, and diterpenoids (S3 File). Fatty acids, alcohols, α,ω-dicarboxylic acids, and hydroxy acids form from the degradation of suberin, a biopolymer found in the outer most layer of the bark periderm [45]. The *n*-alkanes are odd-numbered and related to wax components [46, 47]. The diterpenoids include phenolic diterpenoids, such as ferruginol, sempervirol, totarol, and their degradation products, which are viewed as characteristic of *Podocarpaceae* and *Cupressaceae* [23, 28, 48]. Pimaranes, abietanes, and communic acid, which are found indiscriminately in conifer species [23], were also identified.

### 3.1 Taxonomic differentiation

While species-specific research is limited, the different families are commonly identified by the diterpenoids characteristic of pine species, including specifically phenolic diterpenoids [23, 28]. This section describes the molecular signature of the trees according to the plant taxonomy on the genus and species level using qualitative analysis.

#### 3.1.1 Afrocarpus

**Fig 2:**
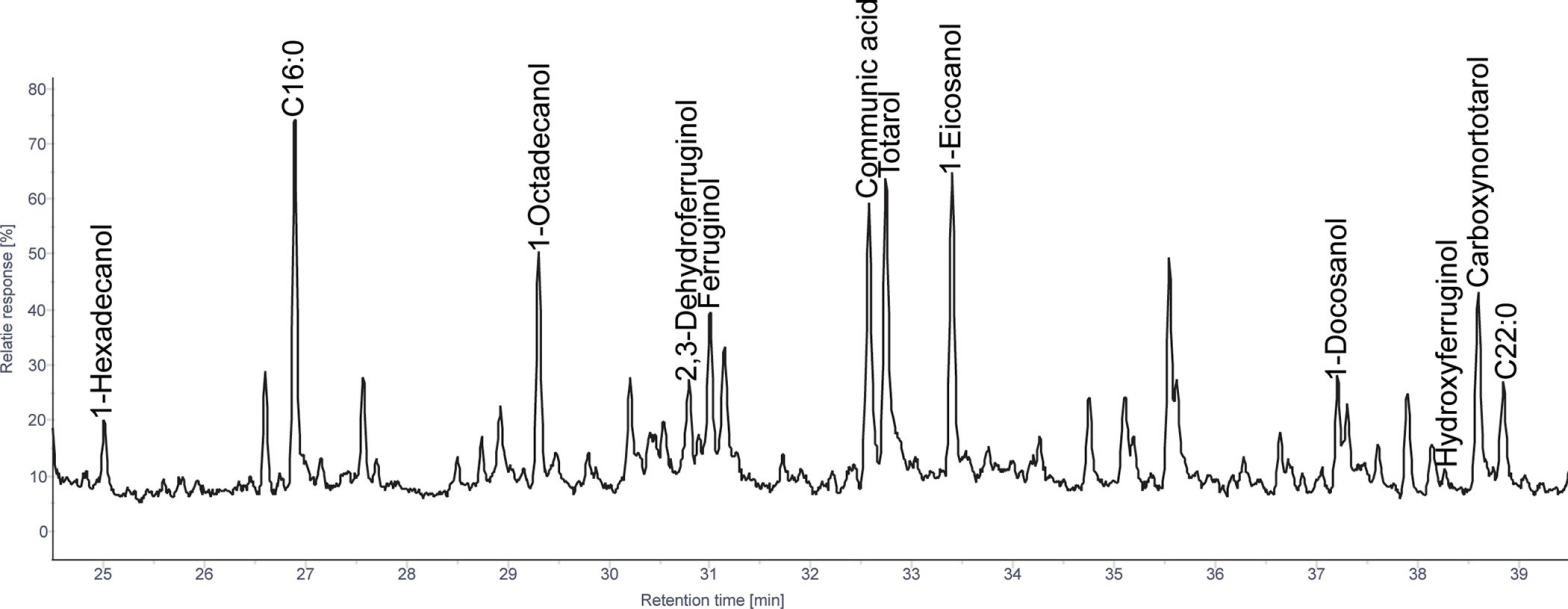
Patial ion chromatogram displaying molecules identified as TMS derivatives from solvent extracted tars produced from *A*. *falcatus* wood.

The identified diterpenoids are primarily phenolic diterpenoids and pimaranes (Fig 2), with the abundances varying between samples. The most abundant phenolic diterpenoids are ferruginol, sempervirol, and totarol, with lesser amounts of 2,3-dehydroferruginol, sugiol, totarane ketones, dehydrototarol, hydroxytotarol, and carboxynortotarol. Pimaranes include high amounts of sandaracopimaric acid, isopimaric acid, and an unknown pimarane (characterized by a base peak at *m/z* 241), with lower amounts of pimaric acid. Traces of kaur-16-ene, abietatriene, and communic acid were also identified.

#### 3.1.2 Podocarpus

**Fig 3:**
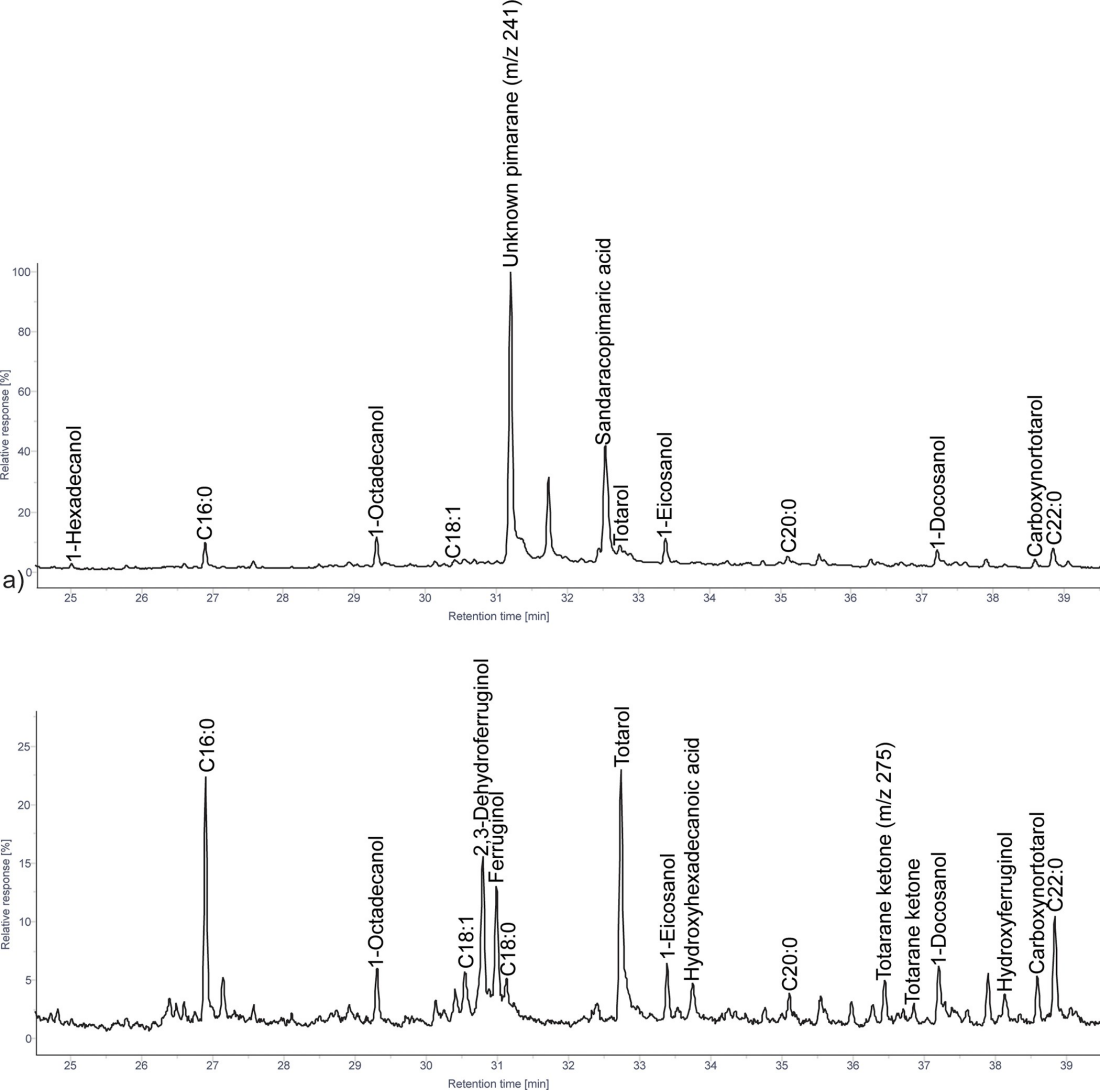
Patial ion chromatogram displaying molecules identified as TMS derivatives from solvent extracted tars produced from *Podocarpus* wood: a) *P*. *henkelii*; b) *P*. *latifolius*.

The diterpenoids consist of phenolic diterpenoids, pimaranes, abietanes, and communic acid (Fig 3). Some variation in terms of the most abundant molecules was noted between the species. *P*. *elongatus* tar and wood contain high amounts of phenolic diterpenoids, with only trace amounts of pimaranes and abietanes. This includes primarily 2,3-dehydroferruginol, totarol, and totarane ketones, with lesser amounts of sempervirol and other derivatives. *P*. *latifolius* tar and wood similarly contains high amounts of totarol, with lower abundances of 2,3-dehydroferruginol, ferruginol, sempervirol, sugiol and other totarane ketones, hydroxyferruginol, and carboxynortotarol (Fig 3B). Within the *Podocarpus* genus, *P*. *henkelii* stands out as unique. Unlike the others, it contains high abundances of pimaranes, including, pimaric acid, sandaracopimaric acid, isopimaric acid, and the unknown pimarane (Fig 3A). Phenolic diterpenoids, including sempervirol, totarol, hydroxytotarol, and carboxynortotarol, were found only in trace amounts in the tar produced from the branches and leaves. Kaur-16-ene was also identified in low abundances.

#### 3.1.3 Widdringtonia

**Fig 4:**
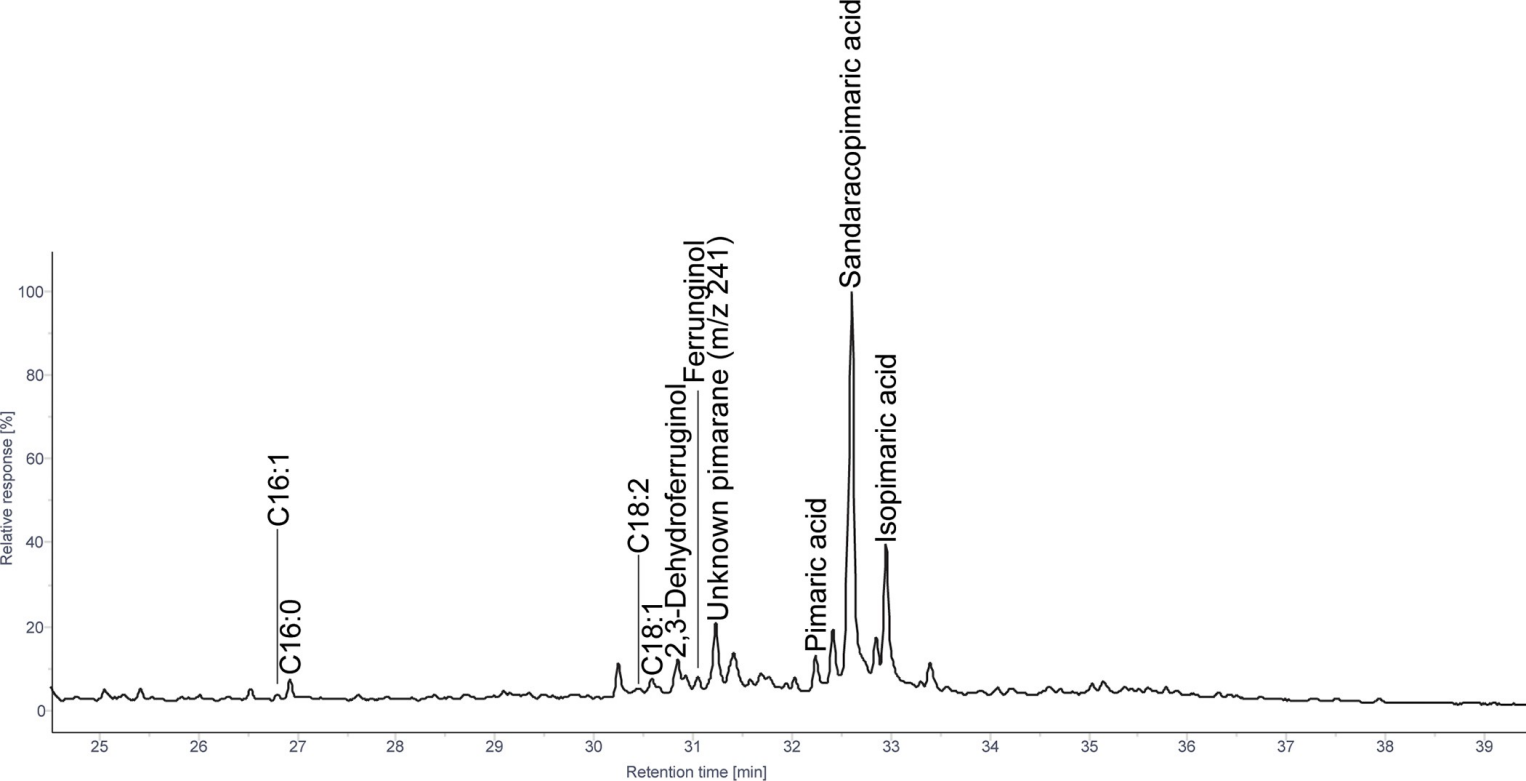
Patial ion chromatogram displaying molecules identified as TMS derivatives from solvent extracted tars produced from *W*. *nodiflora* wood.

In contrast to *Afrocarpus* and *Podocarpus*, both *W*. *cedarbergensis* and *W*. *nodiflora* contain high abundances of pimaranes, namely sandaracopimaric acid, with lesser amounts of pimaric acid, isopimaric acid, and the unknown pimarane (Fig 4). Traces of phenolic diterpenoids in *W*. *cedarbergensis* are restricted to sempervirol and in *W*. *nodiflora* to 2,3-dehydroferruginol and ferruginol.

### 3.2 Material differentiation

Wood, tar produced from branches, tar produced from *Podocarpaceae* leaves, and unmodified resin collected manually from *Widdringtonia* bark and *Podocarpus* seed cones were analyzed. The materials were differentiated primarily based on fatty acids, α,ω-dicarboxylic acids, hydroxy acids, alcohols, and *n*-alkanes. Because the previous section presents an overview of the diterpenoid signature, this section will only touch on the diterpenoids that are indicative of specific raw material.

#### 3.2.1 Wood

Wood was analyzed from *Afrocarpus* (*A*. *falcatus*), *Podocarpus* (*P*. *elongatus*, *P*. *henkelii*, and *P*. *latifolius*), and *Widdringtonia* (*W*. *cedarbergensis* and *W*. *nodiflora*) species. Due to sampling constraints, all wood was collected from young green branches. The samples from *Afrocarpus* and *Podocarpus* species contain saturated fatty acids ranging from C_7:0_–C_24:0,_ maximizing typically at C_16:0_. Unsaturated fatty acids include C_18:1_ and C_18:2_. These general distributions match the tars described below. Traces of even-numbered alcohols were in the samples from most species: 1-octadecanol, 1-eicosanol, 1-docosanol, 1-tetracosanol, and 1-triacontanol. In addition, high amounts of 10-nonacosanol, a secondary alcohol, were in all samples. This is commonly identified in other *Pinaceae* trees, related often to smoke [49–51]. Finally, unexpectedly trace amounts of odd-numbered *n*-alkanes were identified in every species including pentacosane, heptacosane, nonacosane, and triacontane, maximizing at nonacosane. This is unusual because odd-numbered *n*-alkanes are associated with leaf wax [46, 47], and while the reason for their presence is unclear, it may be associated with the type of wood sampled–young green branches. The wood samples from *Widdringtonia* species contrast; these molecule types are rare or entirely absent, with only the above described terpenoids identified.

#### 3.2.2 Tar from branches

**Fig 5:**
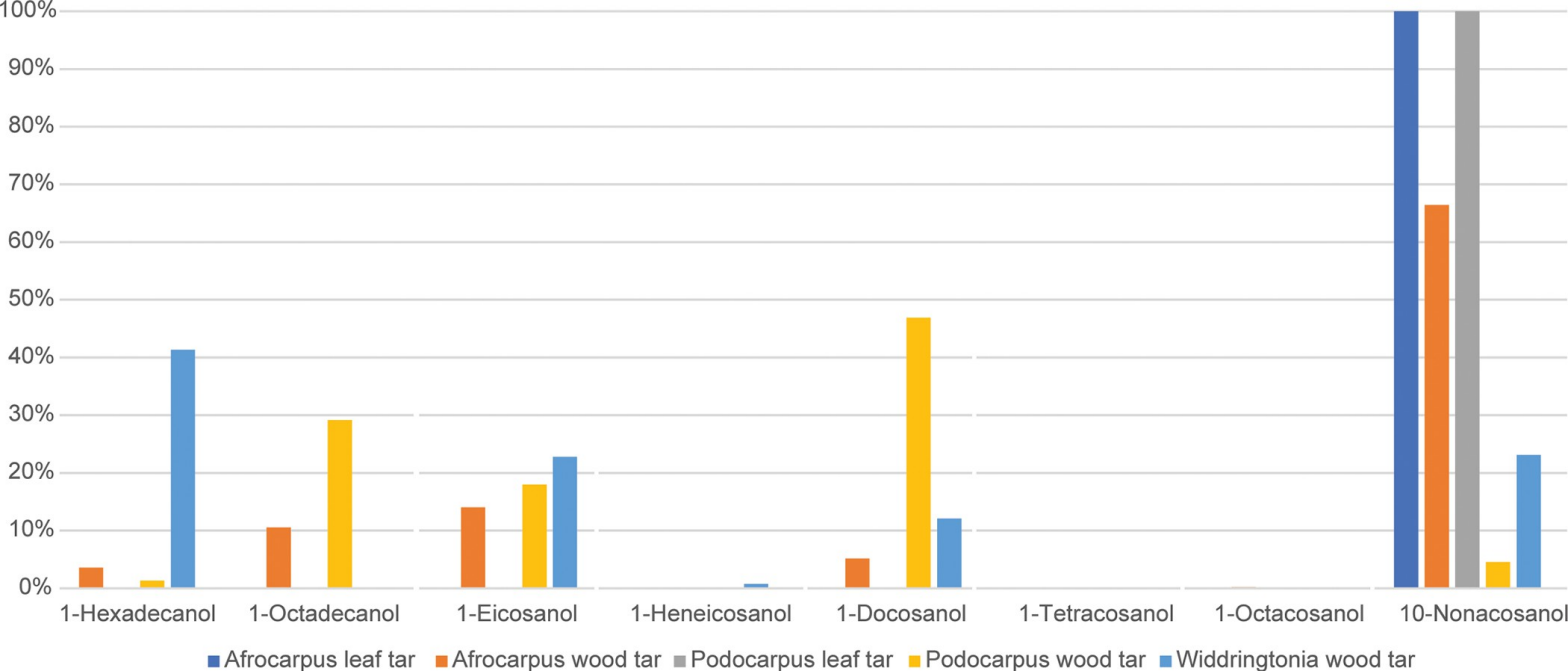
Bar plot of the average relative abundance of the identified alcohols in *Afrocarpus* leaf and wood tar, *Podocarpus* leaf and wood tar, and Widdringtonia wood tar.

Tar made from branches bearing bark was analyzed from *Afrocarpus* (*A*. *falcatus*), *Podocarpus* (*P*. *elongatus*, *P*. *henkelii*, and *P*. *latifolius*), and *Widdringtonia* (*W*. *cedarbergensis* and *W*. *nodiflora*) species. While there are, as noted above, differences in the terpenoids between the genera, there are some overarching shared patterns. The saturated fatty acids range from C_7:0_–C_24:0,_ maximizing most commonly at C_16:0_, with additional high amounts of long-chain even-numbered saturated fatty acids. Unsaturated fatty acids include C_16:1_, C_18:1_, C_18:2_, and C_20:1_, maximizing at C_18:1_. In addition, in each genus, small amounts of α,ω-dicarboxylic acids and hydroxy acids were identified. The α,ω-dicarboxylic acids range from C_5_–C_18_, with all long-chain α,ω-dicarboxylic acids even-numbered; hydroxy acids are even-numbered and have 16–20 carbon atoms, including saturated and unsaturated monomers. Small amounts of even-numbered primary alcohols were present, ranging from C_16_–C_28_, maximizing most commonly at C_22_ (Fig 5). High amounts of 10-nonacosanol were also in tars produced from *A*. *falcatus*, *P*. *henkelii*, *P*. *latifolius*, and *W*. *cedarbergensis* (Fig 5). *N*-alkanes were absent from nearly every tar, excluding *P*. *latifolius*. These include C_27_, C_29_, and C_31_ alkanes. The *n*-alkanes are unusual as they are generally found only in high amounts in tars made from leaves (see Section 3.2.3), and their presence is most likely related to the use of young green shoots to form the tar as these were also shown to contain *n*-alkanes.

#### 3.2.3 Tar from leaves

Tars produced from leaves were tested from *A*. *falcatus*, *P*. *latifolius*, and *P*. *henkelii*. Identified fatty acids range from C_7:0_–C_24:0,_ maximizing at C_16:0_. Some variation is noted between the genera with *Afrocarpus* containing more longer-chain even-numbered fatty acids than *Podocarpus*. Unsaturated fatty acids include C_16:1_, C_18:1_, and C_18:2_, with higher amounts of C_18:1_. Only traces of hydroxy acids were identified (C_16:1_ and C_16:0_). The only alcohol was 10-nonacosanol (Fig 5). Odd-numbered *n*-alkanes were also present; in *A*. *falcatus*, this is restricted to just nonacosane, but in the *Podocarpus* species, there is heptacosane, nonacosane, and triacontane, maximizing at nonacosane.

#### 3.2.4 Resin

The resin was carefully scraped from the bark of *W*. *nodiflora* to exclude any molecular interference from the bark. As such, fatty acids, alcohols, and *n*-alkanes are entirely absent. Only pimaranes were identified, including sandaracopimaric acid and lesser amounts of pimaric acid, isopimaric acid, and the unknown pimarane.

#### 3.2.5 Seed cones

Resinous material from within the seed cones was analyzed from two *Podocarpus* species: *P*. *elongatus* and *P*. *latifolius*. As with the resin scraped from the bark, these contain no fatty acids, alcohols, and *n*-alkanes because there was no suberin, cutin, or wax in the sample. The diterpenoids differ between the two species. From the *P*. *elongatus* cone, kaur-16-ene, communic acid, and pimaranes (pimaric acid and isopimaric acid) were identified. In contrast, only phenolic diterpenoids (2,3-dehydroferruginol, totarol, and a totarane ketone) were identified in the *P*. *latifolius* cone.

## 4. Discussion

Adhesives in South Africa were identified dating as far back as nearly 60,000 BP [4]. Limited organic ingredients are referenced in relation to adhesive production in the Middle and Later Stone Age, including *Podocarpus* resin and tar, beeswax, and other plant exudates [52]. This narrow array is unusual as South Africa is home to over 20,000 plant species [53], and ethnography of southern Africa shows that many of these can be exploited for their adhesive properties [54–56].

The systematic chemical analysis of modern reference material can expand our knowledge of this diverse biome and allow us to understand the use of organics more accurately in the South African archaeological record. At present, lipid residue analysis studies on South African archaeological material are increasing although still uncommon [4–8, 57–62], and a framework for understanding these results is lacking. This dilemma is highly apparent in the study of Stone Age adhesives because most experimental work and reference material target ingredients available in Europe, such as birch tar and pine resin [36]. This issue is compounded by a lack of appropriate ethnographic parallels from South Africa and the Cape region specifically, where we see the majority of archaeological research programs. In contrast, much of the ethnographic research focuses on more arid regions in southern Africa that provide a different array of plant species to exploit [54–56]. The discussion of South African ancient adhesives therefore is still in its early days of research, revolving around a few specific species of plants that have already been chemically characterized in archaeological contexts and ignoring the possibility that other plants may have similar molecular signatures. Refining our ability to correctly identify the used species and material is vital to reconstruct the past reliably, particularly in the case of *Afrocarpus*, *Podocarpus*, and *Widdringtonia* based adhesives because there are substantial differences in raw material procurement and adhesive production. As a step toward ameliorating this situation, the current study targets the chemical profile of these genera. The results contribute to archaeological research and address two primary questions: what trees were exploited by Stone Age populations to produce adhesives and what parts of the trees did people use?

### 4.1 What tree taxa were exploited?

Based on the modern reference collection, attributing adhesives to a species or genus is complicated. *Podocarpaceae* adhesives can be tentatively distinguished by high amounts of phenolic diterpenoids and pimaranes, and specifically a *Podocarpus*-based adhesive can be suggested when there are exclusively phenolic diterpenoids as pimaranes are more characteristic of *Afrocarpus*. *Widdringtonia* differs with only traces of phenolic diterpenoids and high amounts of pimaranes. The situation, however, is complicated by the differential preservation of diterpenoids. Pimaranes, which are essential to classifying *Widdringtonia*, lack a conjugated double bond, making them susceptible to degradation [63]. Caution must be applied when using biomarkers that are not stable to interpret lipid origins [64]. The degradation of pimaranes could make a *Widdringtonia* based adhesive appear like a *Podocarpaceae* adhesive, while an *Afrocarpus* adhesive can appear to be a *Podocarpus* adhesive. Therefore, in the total absence of pimaranes, concrete identification should be avoided. Considering this, we raise the need for a reassessment of identified archaeological adhesives.

Prior GC-MS studies on South African ancient adhesives identified the use of *Podocarpaceae* tar or resin (Table 3) dating as far back as the Middle Stone Age (MSA) at Diepkloof Rock Shelter [4]; it continued to be used through the Later Stone Age (LSA) at Elands Bay Cave, Melkhoutboom Cave, and Steenbokfontein Cave [5–7, 65, 66]. This use of *Podocarpaceae* is suggested at these four sites based on the presence of phenolic diterpenoids. In Diepkloof Rock Shelter, Elands Bay, and Border Cave, *P*. *elongatus* was suggested as a likely source based on comparison to one modern reference and the link to the archaeological botanic remains at these sites [4–6, 12]. However, based on the chromatograms from these studies, most peaks were unidentified, and the defined molecules can be found in *Afrocarpus*, *Podocarpus*, and *Widdringtonia* genera, deterring confident identification. A more cautious approach was taken for the Melkhoutboom residues for which *Afrocarpus* and *Podocarpus* were listed as possible sources [7]. In all these examples, the absence of pimaranes and abietanes is worrying, suggesting an advanced stage of degradation that prohibits identification to the genus *Podocarpaceae* despite the abundance of phenolic diterpenoids. An even broader interpretation of *Podocarpaceae* or *Cupressaceae* resin was given for Steenbokfontein [65, 66], in which only phenolic diterpenoids were identified. In all these examples, the primary diterpenoids identified are totarane ketones, which were found in low abundances in most of the modern reference material. Ketones can be synthesized from other molecules through several pathways, including oxidation [67–69], and metals in the soil can also act as a catalyst for this process [70–72], so the abundance of totarane ketones in archaeological examples most likely relates to degradation processes. Accelerated aging studies however are required to support this.

**Table 3:** Overview of the archaeological coniferous resin and tar in South Africa^1^.

Site	Extraction method	Molecules identified		Original interpretation	Current study’s interpretation	Reference
**Border Cave (LSA)**	Saponification	**Diterpenoids**	• Sugiol • Totarane ketones	*Podocarpaceae* (likely *P*. *elongatus*) tar	*Podocarpaceae*/*Widdringtonia* tar from wood	[6]
		**α,ω-Dicarboxylic acids**	• C_7_–C_12_, C_16_, C_18_, C_20_–C_22_			
		**Hydroxy acids**	• Hydroxy C_8_–C_16_, C_18_, C_20_, C_22_ • Dihydroxy C_18_			
**Diepkloof Rock Shelter (MSA)**	Solvent extraction	**Diterpenoids**	• Sugiol • Totarane ketones	*Podocarpaceae* (likely *P*. *elongatus*) oxidized resin	*Podocarpaceae*/*Widdringtonia* resin/tar	[4]
		**α,ω-Dicarboxylic acids**	• C_7_–C_14_			
		**Alcohols**	• Unspecified			
		**Alkanes**	• C_20_-C_35_			
**Elands Bay Cave (LSA)**	Solvent extraction	**Diterpenoids**	• 3-Ketototarol • 4-Carboxy, 7-ketototarol • Totarol	*Podocarpaceae* (likely *P*. *elongatus*) resin	*Podocarpaceae*/*Widdringtonia* resin/tar	[5]
		**α,ω-Dicarboxylic acids**	• C_9_			
		**Alcohols**	• C_14_–C_18_			
**Melkhoutboom Cave (LSA)**	Saponification	**Diterpenoids**	• 2,3-dehydroferruginol • Dehydrosempervirol • Dehydrototarol • Totarol • Sempervirol • Sugiol • Totarane ketones	*Podocarpaceae* tar	*Podocarpaceae*/*Widdringtonia* tar from wood	[7]
		**α,ω-Dicarboxylic acids**	• C_9_, C_16_, C_18_, C_20_, C_22_			
		**Hydroxy acids**	• Hydroxy C_16_, C_18_, C_20_, C_22_			
		**Diterpenoids**	• 7-Oxodehydroabietic acid • Dehydroabietic acid • Sugiol • Totarane ketones	*Widdringtonia* tar	*Widdringtonia* tar	
		**α,ω-Dicarboxylic acids**	• C_16_, C_22_			
		**Hydroxy acids**	• Hydroxy C_16_, C_18_, C_22_			
**Renbann Cave (LSA)**	Saponification	**Diterpenoids**	• 7-Hydroxydehydroabietic acid • 8,15-Pimaradien-18-oate • Dehydroabietic acid • Ferruginol	*Widdringtonia* tar	*Widdringtonia* tar	[7]
		**α,ω-Dicarboxylic acids**	• C_16_, C_18_			
		**Hydroxy acids**	• Hydroxy C_16_, C_18_, C_22_			
		**Alcohols**	• C_22_, C_24_, C_26_, C_28_, C_30_			
		**Alkanes**	• C_27_, C_29_, C_31_			
**Sibudu Cave (MSA and LSA)**	Saponification	**Diterpenoids**	• 15-Hydroxy-7-oxodehydroabietic acid • 7-Oxodehydroabietic acid • Dehydroabietic acid • Didehydroabietic acid • Isopimaric acid	Conifer/*Podocarpus* resin	Conifer tar	[8]
		**α,ω-Dicarboxylic acids**	• C_5_–C_7_, C_12_, C_13_			
		**Hydroxy acids**	• Hydroxy C_7_, C_9_, C_12_, C_13_, C_16_ • Dihydroxy C_18_			
		**Alcohols**	• C_16_, C_18_			
**Steenbokfontein Cave (LSA)**	Saponification	**Diterpenoids**	• 14-isopropylpodocarpa-8,11,13-triene-7,13-diol • 2,3-Dehydroferruginol • Dehydrototarol • Sempervirol • Sugiol • Totarane ketones • Totarol	*Podocarpaceae*/*Widdringtonia*	*Podocarpaceae*/*Widdringtonia* tar	[65, 66]
		**α,ω-Dicarboxylic acids**	• C_16_, C_18_			
		**Hydroxy acids**	• Hydroxy C_6_, C_7_, C_16_, C_18_, C_22_ • Dihydroxy C_6_, C_8_ • Trihydroxy C_18_			

^1^ This table does not include all organic constituents identified from adhesives from these sites and displays only those potentially related to *Podocarpaceae* and *Widdringtonia* resin and tar.

An adhesive formed from *Widdringtonia* was suggested at the Later Stone Age sites of Melkhoutboom Cave and Renbaan Cave (Table 3) based on the abundance of pimaranes and abietanes in favor of phenolic diterpenoids [7]. These results are more in line with our study. While some *Podocarpus* and *Afrocarpus* samples do contain high abundances of pimaranes and abietanes, this is always paired with high amounts of phenolic diterpenoids. In the *Widdringtonia* samples, there are always high abundances of pimaranes and abietanes and only traces of phenolic diterpenoids. At Melkhoutboom and Renbaan caves, no archaeobotanical remains were recovered that could support the results. However, based on the known distribution of *Widdringtonia*, concentrated in primarily mountainous regions [38], these trees were likely present, particularly at Melkoutboom Cave, which is located in the Cape Folded Mountain Belt. Unusually, *W*. *cedarbergensis* charcoal was recovered from Diepkloof Rock Shelter [11], but there is no evidence that the occupants exploited it for adhesive production.

At Sibudu Cave, an attempt to differentiate the genera based on lipid signature was not made as it contained only abietanes and pimaranes; a conifer resin was suggested [8, 52]. Despite this the adhesive was still connected to *Podocarpus* based on the charcoal remains at the site [8]. Caution should be applied here because the molecular signature can also be connected to *Afrocarpus* and *Widdringtonia* or even a different conifer species.

To summarize, based on the modern reference collection, we propose that several archaeological GC-MS studies overinterpreted biomarkers that can have multiple origins and too narrowly assigned a residue source. While suggestions for a *Podocarpaceae*-based adhesive can be made for both the Middle and Later Stone Age (across several regions of South Africa), a specific genus or species cannot be confirmed. *Widdringtonia* appears to have a punctuated appearance, having been identified at two coastal sites during the final Later Stone Age. A cautious approach that relies first and foremost on a comprehensive GC-MS reference collection and then supports the results with archaeobotanical remains and environmental context is appropriate and should be a standard practice in adhesive identification.

### 4.2 What materials were exploited?

Recent work focuses on the production of tar from *Podocarpus* leaves. The leaves were suggested as an appropriate source for adhesive production because they contain high amounts of resin in comparison to the bark, and when processed in certain ways, the leaves can be used to form a strong adhesive [3]. To test this hypothesis against archaeological material, differences in the molecular signature of adhesives made from leaves and bark must first be identified.

Tars formed from *Afrocarpus* and *Podocarpus* leaves contain diterpenoids similar to tar produced from the bark of *Afrocarpus* and *Podocarpus* trees. Based on diterpenoids alone, the source cannot be distinguished. However, when the wider molecular signature is analyzed, the leaves can be distinguished based on the presence of odd-numbered *n*-alkanes, which are characteristic of plant wax [46, 73]. These are nearly entirely absent in tar produced from branches. Instead, this tar contains long-chain α,ω-dicarboxylic acids, hydroxy acids, and even-numbered alcohols. While these can be found individually in other materials naturally [74–79], the combination of these three is indicative of tar formed from bark as these are degradation products of suberin [45, 80]. α,ω-Dicarboxylic acids and hydroxy acids were not identified in unaltered wood samples. However, this may be explained by differences in extraction methods; α,ω-dicarboxylic acids and hydroxy acids were primarily identified in the reference material extracted using saponification, and it is possible that these could appear in other materials if extracted differently. “Pure” resins extracted from the bark and seed cones contain only diterpenoids.

Applying this knowledge to published archaeological material is difficult as the discussion is centered around the terpenoids, and often other molecule types are only discussed in brief (Table 3). Further, several of the molecule types that are indicative of the material exploited can be found in other materials, namely other plant waxes, degraded beeswax, and sediment, mandating caution in interpretation [64] as these could represent mixtures with other products. For example, at Diepkloof Rock Shelter, *n*-alkanes with C_20_–C_35_ (maximizing at C_27_) were present, with only a strong odd over even abundance [4], similar to tar produced from leaves. However, these can also be interpreted as resulting from sediment contamination, based on the identification of odd- and even-numbered *n*-alkanes [81], or a wax or additional plant material [47, 82, 83] based on the combined presence of *n*-alkanes and unspecified alcohols and esters [4]. Such a mixture is even clearer at Renbaan Cave. A sample with evidence for *Widdringtonia* resin contained odd-numbered *n*-alkanes (C_27_, C_29_, and C_31_) [7]; our study shows that these are not found naturally in *Widdringtonia*, and because they were paired with long-chain even-numbered saturated hydroxy fatty acids and alcohols, they were interpreted as related to beeswax [7]. Only when there are exclusively diterpenoids and odd-numbered *n*-alkanes can tar formed from leaves be confidently identified. Therefore, from the currently characterized examples of *Afrocarpus* and *Podocarpus*, there is no clear evidence of tar produced from leaves. On the contrary, archaeological examples with *n*-alkanes may relate to the production of a compound adhesive.

Some examples, however, from Border Cave, Melkhoutboom Cave, Renbaan Cave, and Steenbokfontein Cave contain phenolic diterpenoids paired with α,ω-dicarboxylic acids and hydroxy acids [6, 7, 65, 66], and these can be considered more indicative of tar produced from bark. It must be noted that in most cases where the adhesive was identified as a tar (Table 3), the samples were saponified, a process that transesterifies suberin into its core components [84–86]. Because these were uncommon in samples that were not saponified, the adhesive was often identified as a resin, as at Diepkloof Rock Shelter and Elands Bay Cave [4, 5]. More accurately, without saponification, no attempt should be made to differentiate between tar and resin. Saponification, however, is not without its short comings, deterring the identification of wax esters and acylglycerols, and as such, interpretations can be complicated when mixtures are present. In the case of South African archaeological contexts where organic admixtures were identified [4–7], multiple extraction methods are called for to discern between the use of tar versus resin and additives, and even further elucidation can be achieved through the use of other mass spectrometry, microscopy, and spectroscopy techniques.

## 5. Conclusion

This study forms one of the most comprehensive reference databases of the molecular profile of specific conifers native to South Africa, including 26 samples from six species and five different materials from trees from the *Afrocarpus*, *Podocarpus*, and *Widdringtonia* genera. The results enhance our understanding of conifer-based adhesive production in the South African archaeological record, setting guidelines for genus and raw material identification.

While *Podocarpus* resin and tar is frequently cited as a key ingredient in adhesive production [4–8], based on the reference material, *Podocarpus*, *Afrocarpus*, and *Widdringtonia* are difficult to distinguish especially when preservation is considered. Modern *Podocarpaceae* contains high amounts of phenolic diterpenoids and, in the case of *Afrocarpus*, pimaranes, while *Widdringtonia* contains trace amounts of phenolic diterpenoids and high amounts of pimaranes. Once degraded, these may appear similar. When these patterns are applied with caution to the archaeological record, many of the previously archaeologically identified *Podocarpus* resins are more accurately classified as *Podocarpaceae* or are too degraded to specify, and no genus or species-level identification should be given. Accelerated aging studies are recommended as a next step in elucidating the differences between archaeological samples and modern reference material, and these may provide insight on oxidation processes and further reasoning behind observed discrepancies.

A clearer divide can be proposed for materials used in adhesive production. While diterpenoids are essential to identify tree species, differentiating tar produced from leaves and wood relies on other molecule types, namely *n*-alkanes, α,ω-dicarboxylic acids, hydroxy acids, and alcohols. Leaves of *Afrocarpus* and *Podocarpus* species were suggested as an ideal matrix for tar production [3], and the tar can be distinguished by a high amount of long-chain odd-numbered *n*-alkanes found in the leaf wax. In contrast, an adhesive produced from the bark of either *Afrocarpus*, *Podocarpus*, or *Widdringtonia* contains α,ω-dicarboxylic acids, hydroxy acids, and alcohols, formed from the degradation of suberin, and no *n*-alkanes. Based on these definitions, at present, there is no definitive archaeological evidence for tar production using leaves. However, suggestions can be made for wood-based tar production when adhesive samples are saponified and for mixtures of tar/resin with beeswax when adhesives are solvent extracted. The resin from the seed cones of *Afrocarpus* and *Podocarpus* has yet to be considered in literature as a potential ingredient in archaeological adhesive production, and more testing is required to elucidate its molecule profile as well as the most efficient way to extract and transform it into a useable adhesive. However, based on the current samples, the resin from the seed cones contains exclusively diterpenoids, making them likely indistinguishable from degraded tar and resin samples.

In reviewing the archaeological record of adhesive production in South Africa, this study shows how having an extensive reference collection is essential for interpreting the use of organics in the past. By using a small and unrepresentative reference collection, the potential for misinterpretation is great. This reference collection, while thorough and encompassing a range of taxa (from multiple locations), instrumentation, and extraction protocols, demonstrates that differentiating between tree species is complicated because the lipid signatures are not perfectly consistent. It is unclear if increasing the sample size further would create a more representative average. Therefore, when interpreting the use of conifers in adhesive production in South Africa based on molecular analysis, we propose that caution must be applied to avoid overgeneralizations, and the results from molecular studies should be viewed considering the archaeological record (*e*.*g*. pollen, charcoal, and other macro- and micro-botanical remains) as well as the environmental landscape.

## Supporting information

S1 File Expanded material and methods. (DOCX)

S2 File Mass spectra of diterpenoids discussed in this paper. (DOCX)

S3 File Overview of the molecules identified in this paper and the abundance of diterpenoids and alcohols. (XLSX)
