# Supplementary material for: Podocarpaceae and Cupressaceae: A tale of two conifers and ancient adhesives production in South Africa
Source: PLoS One. 2024 Nov 13;19(11):e0306402. doi: 10.1371/journal.pone.0306402 (PMC11560044; doi:10.1371/journal.pone.0306402)
Supplement: S1 File — (DOCX) [file pone.0306402.s001.docx]

**Expanded materials and methods**

Specimens from four species of *Podocarpaceae* and two species of *Cupressaceae* were globally sourced (Table 1). This includes *A. falcatus*, *P. elongatus, P. henkelii*, *P. latifolius*, *W. cedarbergensis*, and *W. nodiflora*. The tar was produced and analyzed in three methods at TU Delft. the University of Pisa, and the University of Oxford (Table 1).

**TU Delft**

The tars analyzed at TU Delft were produced using 10 g of leaves or branches bearing bark. These were placed in a glass test tube fitted with a silicone stopper and a glass tube, leading to a cooled collection beaker. The temperature was recorded using thermocouples placed in the test tube in the middle of the leaves and in the cooled collection vessel. A propane torch was used to heat the material for 11–18 minutes until no more tar was produced. All samples were heated to a maximum temperature of approximately 550℃. In addition, unaltered samples of resin from Widdringtonia bark and *Podocarpus elongatus* (purchased from Silverhill Seeds; Cape Town, South Africa) and *latifolius* seed cones (collected from Pinetum Blijdenstein; Hilversum, Netherlands) were collected for analysis.

Following established protocols [1], 4 mL of dichloromethane (DCM) (HPLC grade) was added to sub-samples (ca. 4 mg), and the lipids were extracted by ultrasonication (30 minutes). An aliquot was dried under a gentle stream of nitrogen and derivatized using 50 μL bis(trimethysilyl)trifluoroacetamide containing 1% trimethylchlorosilane, 10 μL of DCM, and 5 µL of pyridine. The reaction took place at room temperature for 20 minutes. The samples were then dried under nitrogen stream at 30 °C and rehydrated with DCM.

GC-MS analysis was performed on an Agilent 7890B GC coupled with an Agilent 5977B EI MSD interface and a FID. The GC was fitted with a nonpolar Agilent J&W DB5 MS column (30 m × 0.25 mm i.d.; 0.25 μm film thickness). The samples were introduced in splitless mode at 300 °C with a septum purge flow of 3mL min^-1^. The oven temperature was held isothermally for 2 min at 50 °C, ramped at 10 °C min^-1^ to 150 °C and 4°C min^-1^ to 320 °C, and then held at 320 °C for 20 minutes. The analysis was carried out using helium as the carrier gas at a constant flow rate of 1.6 mL min^-1^ (average velocity 32.146 cm sec^-1^). The temperature of the FID was set at 340 °C. The hydrogen flow was 30 mL min^-1^, the synthetic air flow was 400 mL min^-1^, and the nitrogen flow was 30 mL min^-1^. The temperatures of the ion source were set at 230 °C and the transfer line at 280 °C. The mass spectrometer was monitored to scan 35–950 *m/z* with an ionizing voltage of 70 eV. The resulting chromatograms were interpreted using the National Institute of Standards and Technology (NIST; version 2.3).

**University of Pisa extraction and analysis protocol**

The tars were made at the University of Pisa and produced by heating ca. 1 g of bark and woody tissue in a purged and sealed glass test tube for 30 minutes in a muffle furnace; the tar was heated to a maximum temperature of approximately 500℃.

Solid fragments of tar were removed by glass pipette and scalpel. Any staining was removed with hexane, dichloromethane and methanol (~600 µL, 3 x 10min). The fractions were combined and dried under a stream of nitrogen. Following this, the samples were saponified following established protocols [2, 3] in a hydroalcoholic solution of 10% wt potassium hydroxide (KOH). The solution was heated at 60°C for 3 hours. Following this, the neutral fraction was extracted with *n*-hexane (400 μL 3x, combined into a single neutral fraction). The remaining solution was acidified to a pH of 2 using 6M HCl. The acid fraction was extracted with 400 μL 3x diethyl ether, combined into a single acid fraction. The neutral and acidic fractions were dried under a stream of nitrogen and silylated. Both the neutral and acid fraction with derivatized using 20 μL bis(trimethysilyl)trifluoroacetamide containing 1% trimethylchlorosilane and 150 µL of isooctane. The reaction took place at 60°C for 30 minutes.

The samples were analyzed using a Trace GC 2000 GC system coupled to an ITQ 900 ion trap (Thermo Fisher, U.S.A.). Samples were injected in splitless mode at 280°C. The GC was fitted with an Agilent J&W HP-5MS column (30 m length × 0.25 mm i.d.; 0.25 μm film thickness; 5% diphenyl/95% dimethylpolysiloxane stationary phase). Chromatographic conditions were as follows: initial temperature 80°C, 2 min isothermal hold, 10°C min^-1^ up to 200°C, 4 min isothermal hold, 6°C min^-1^ up to 280°C, and finally a 40 min isothermal hold [4]. The helium (purity 99.9995%) gas flow was set in constant flow mode at 1.2 mL m^-1^. MS parameters were: electron impact ionization (EI) of 70 eV, ion source temperature 230°C, interface temperature 280 °C, a scan range of 50-700 m/z. The injection volume was 2 μL. Peak assignment for all acquired data was based on comparisons to mass spectra libraries (NIST 11 main EI MS library and a prepared AMDIS library) and published data.

**University of Oxford extraction and analysis protocol**

Wood samples were analyzed at the University of Oxford. One sample (*P. latifolius* wood) was saponified following the method applied at the University of Pisa. All other material underwent solvent extraction by sonication (3 x 10min) utilizing DCM and methanol in order of increasing polarity. These fractions were then combined for downstream analysis, with each aliquot dried under a stream of nitrogen and silylated. Samples were derivatized with 20 µL bis(trimethysilyl)trifluoroacetamide containing 1% trimethylchlorosilane 150 µL of *n*-hexane. The reaction took place at 60°C for 30 minutes.

The samples were analyzed using an Agilent 7820A GC coupled to an Agilent 5975 quadrupole MS. The GC was fitted with a Restek Rxi-5ms column (30 m length x 0.25mm i.d.; 0.25 μm film thickness, 5% diphenyl/95% dimethylpolysiloxane stationary phase). Samples were injected in splitless mode at a temperature of 300°C. Chromatographic conditions were one of two protocols: 1) initial temperature 80°C, 2 min isothermal hold, 10 °C/min up to 200 °C, 4 min isothermal hold, 6 C/min up to 280°C, 40 min isothermal (adapted from Ribechini et al. 2009); or, initial temperature 50°C, 2 min isothermal hold, 50-300°C at 10°C/min with a 10 min isothermal hold at 300°C [5]. The helium (purity 99.9995%) gas flow was set in constant flow mode at 1.2 mL min^-1^. MS parameters were: operated in EI ionization mode electron impact ionization (EI) of 70 eV, ion source temperature 280°C, interface temperature 280 °C, and a scan range of 50-650 m/z. The injection volume was 2 μL. Peak assignment for all acquired data was based on comparisons to mass spectra libraries (NIST 11 main EI MS library and a prepared AMDIS library) and published data.

Table 1. List of analyzed samples, their taxonomy, and origin, and the location where the laboratory analysis occured

| **Sample** | **Material** | **Family** | **Genus** | **Species** | **Analysis location** | **Source** |
| --- | --- | --- | --- | --- | --- | --- |
| A. falcatus 1 | Branch tar | *Podocarpaceae* | *Afrocarpus* | *A. falcatus* | Pisa | Royal Botanic Gardens Edinburgh, UK |
| A. falcatus 2 | Branch tar | *Podocarpaceae* | *Afrocarpus* | *A. falcatus* | Pisa | Royal Botanic Gardens Edinburgh, UK |
| A. falcatus 3 | Leaf tar | *Podocarpaceae* | *Afrocarpus* | *A. falcatus* | Delft | Pinetum Blijdenstein, Hilversum, Netherlands |
| A. falcatus 4 | Branch tar | *Podocarpaceae* | *Afrocarpus* | *A. falcatus* | Delft | Pinetum Blijdenstein, Hilversum, Netherlands |
| A. falcatus wood 1 | Wood | *Podocarpaceae* | *Afrocarpus* | *A. falcatus* | Oxford | Royal Botanic Gardens Edinburgh, UK |
| A. falctatus wood 2 | Wood | *Podocarpaceae* | *Afrocarpus* | *A. falcatus* | Oxford | Royal Botanic Gardens Edinburgh, UK |
| P. elongatus 1 | Branch tar | *Podocarpaceae* | *Podocarpus* | *P. elongatus* | Pisa | Royal Botanic Gardens Edinburgh, UK |
| P. elongatus 2 | Branch tar | *Podocarpaceae* | *Podocarpus* | *P. elongatus* | Pisa | Royal Botanic Gardens Edinburgh, UK |
| P. elongatus 3 | Seed cone | *Podocarpaceae* | *Podocarpus* | *P. elongatus* | Delft | Capetown, South Africa |
| P. elongatus wood 1 | Wood | *Podocarpaceae* | *Podocarpus* | *P. elongatus* | Oxford | Royal Botanic Gardens Edinburgh, UK |
| P. henkelii 1 | Branch tar | *Podocarpaceae* | *Podocarpus* | *P. henkelii* | Pisa | Royal Botanic Gardens Edinburgh, UK |
| P. henkelii 2 | Branch tar | *Podocarpaceae* | *Podocarpus* | *P. henkelii* | Pisa | Royal Botanic Gardens Edinburgh, UK |
| P. henkelii 3 | Leaf tar | *Podocarpaceae* | *Podocarpus* | *P. henkelii* | Delft | Pinetum Blijdenstein, Hilversum, Netherlands |
| P. henkelii 4 | Branch tar | *Podocarpaceae* | *Podocarpus* | *P. henkelii* | Delft | Pinetum Blijdenstein, Hilversum, Netherlands |
| P. henkelii wood 1 | Wood | *Podocarpaceae* | *Podocarpus* | *P. henkelii* | Oxford | Royal Botanic Gardens Edinburgh, UK |
| P. henkelii wood 2 | Wood | *Podocarpaceae* | *Podocarpus* | *P. henkelii* | Oxford | Royal Botanic Gardens Edinburgh, UK |
| P. latifolius 1 | Wood | *Podocarpaceae* | *Podocarpus* | *P. latifolius* | Oxford | Capetown, South Africa |
| P. latifolius 2 | Seed cone | *Podocarpaceae* | *Podocarpus* | *P. latifolius* | Delft | Pinetum Blijdenstein, Hilversum, Netherlands |
| P. latifolius 3 | Leaf tar | *Podocarpaceae* | *Podocarpus* | *P. latifolius* | Delft | Pinetum Blijdenstein, Hilversum, Netherlands |
| P. latifolius 4 | Branch tar | *Podocarpaceae* | *Podocarpus* | *P. latifolius* | Delft | Pinetum Blijdenstein, Hilversum, Netherlands |
| W. cedarbergensis 1 | Branch tar | *Cupressaceae* | *Widdringtonia* | *W. cedarbergensis* | Pisa | Royal Botanic Gardens Edinburgh, UK |
| W. cedarbergensis 2 | Branch tar | *Cupressaceae* | *Widdringtonia* | *W. cedarbergensis* | Pisa | Royal Botanic Gardens Edinburgh, UK |
| W. cedarbergensis wood 1 | Wood | *Cupressaceae* | *Widdringtonia* | *W. cedarbergensis* | Oxford | Royal Botanic Gardens Edinburgh, UK |
| W. nodiflora wood 1 | Wood | *Cupressaceae* | *Widdringtonia* | *W. nodiflora* | Oxford | Economic Botany Collection, Kew Gardens, UK |
| W. nodiflora 1 | Resin | *Cupressaceae* | *Widdringtonia* | *W. nodiflora* | Delft | Capetown, South Africa |
| W. nodiflora 2 | Branch tar | *Cupressaceae* | *Widdringtonia* | *W. nodiflora* | Delft | Capetown, South Africa |

**List of references:**

1. Regert M, Alexandre V, Thomas N, Lattuati-Derieux A. Molecular characterisation of birch bark tar by headspace solid-phase microextraction gas chromatography–mass spectrometry: A new way for identifying archaeological glues. Journal of Chromatography A. 2006;1101(1–2):245-53. doi: 10.1016/j.chroma.2005.09.070.

2. Andreotti A, Bonaduce I, Colombini MP, Gautier G, Modugno F, Ribechini E. Combined GC/MS Analytical Procedure for the Characterization of Glycerolipid, Waxy, Resinous, and Proteinaceous Materials in a Unique Paint Microsample. Analytical Chemistry. 2006;78(13):4490-500. doi: <https://doi.org/10.1021/ac0519615>.

3. Colombini MP, Giachi G, Modugno F, Pallecchi P, Ribechini E. The characterization of paints and waterproofing materials from the shipwrecks found at the archaeological site of the Etruscan and Roman harbour of Pisa (Italy *). Archaeometry. 2003;45(4):659-74. doi: <https://doi.org/10.1046/j.1475-4754.2003.00135.x>.

4. Ribechini E, Orsini S, Silvano F, Colombini MP. Py-GC/MS, GC/MS and FTIR investigations on LATE Roman-Egyptian adhesives from opus sectile: New insights into ancient recipes and technologies. Analytica Chimica Acta. 2009;638(1):79-87.

5. Woodworth M, Bernal D, Bonifay M, Garnier N, Keay S, Pecci A, et al. The content of African Keay 25/Africana 3 amphorae: initial results of the CORONAM Project. ArcheoAnalytics: Chromatography and DNA analysis in Archaeology; Esposende, Portugal2015. p. 41-57.
