# Supplementary figures and images for: Podocarpaceae and Cupressaceae: A tale of two conifers and ancient adhesives production in South Africa
Source: PLoS One. 2024 Nov 13;19(11):e0306402. doi: 10.1371/journal.pone.0306402 (PMC11560044; doi:10.1371/journal.pone.0306402)

**Mass spectra of diterpenoids discussed in this paper**


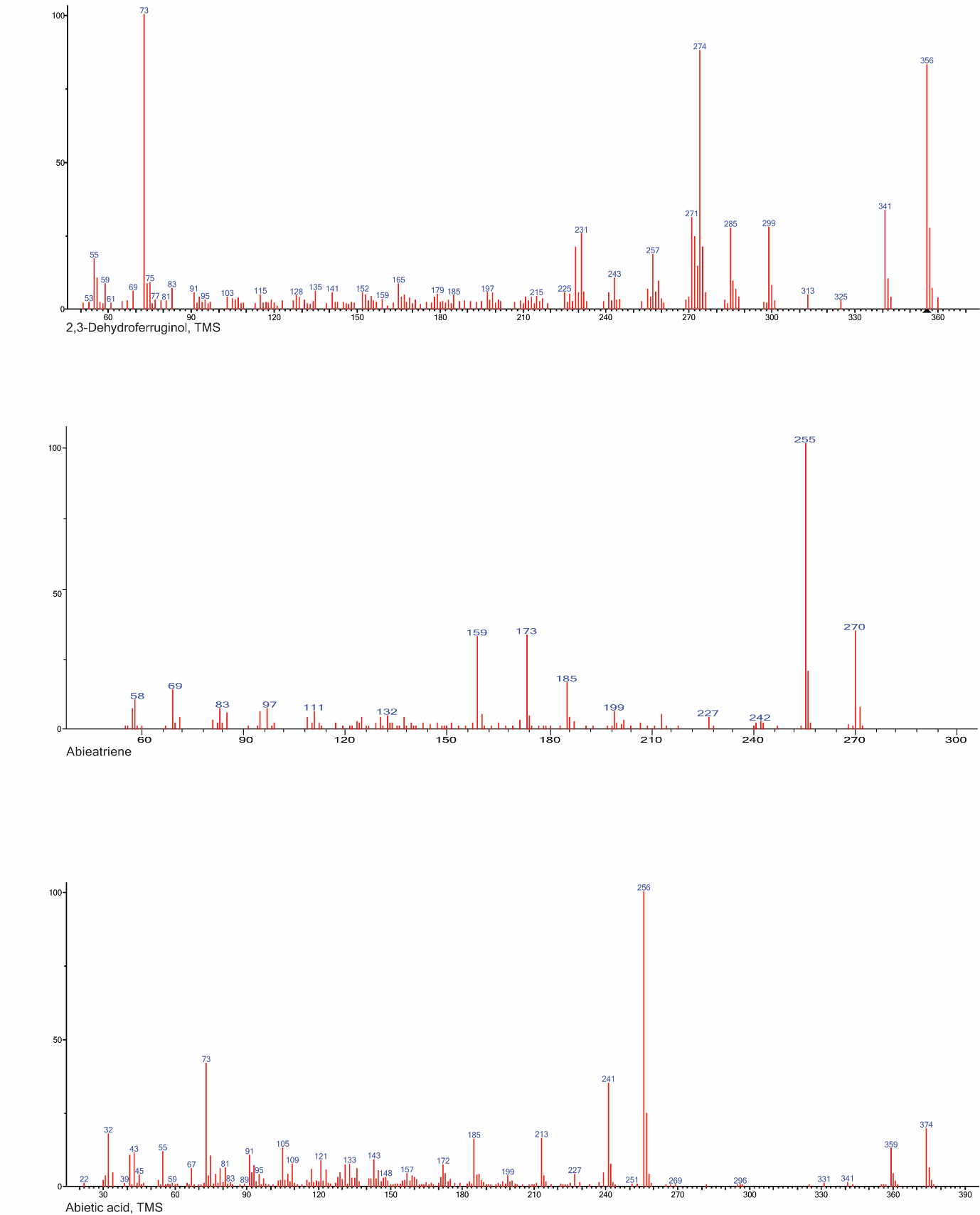


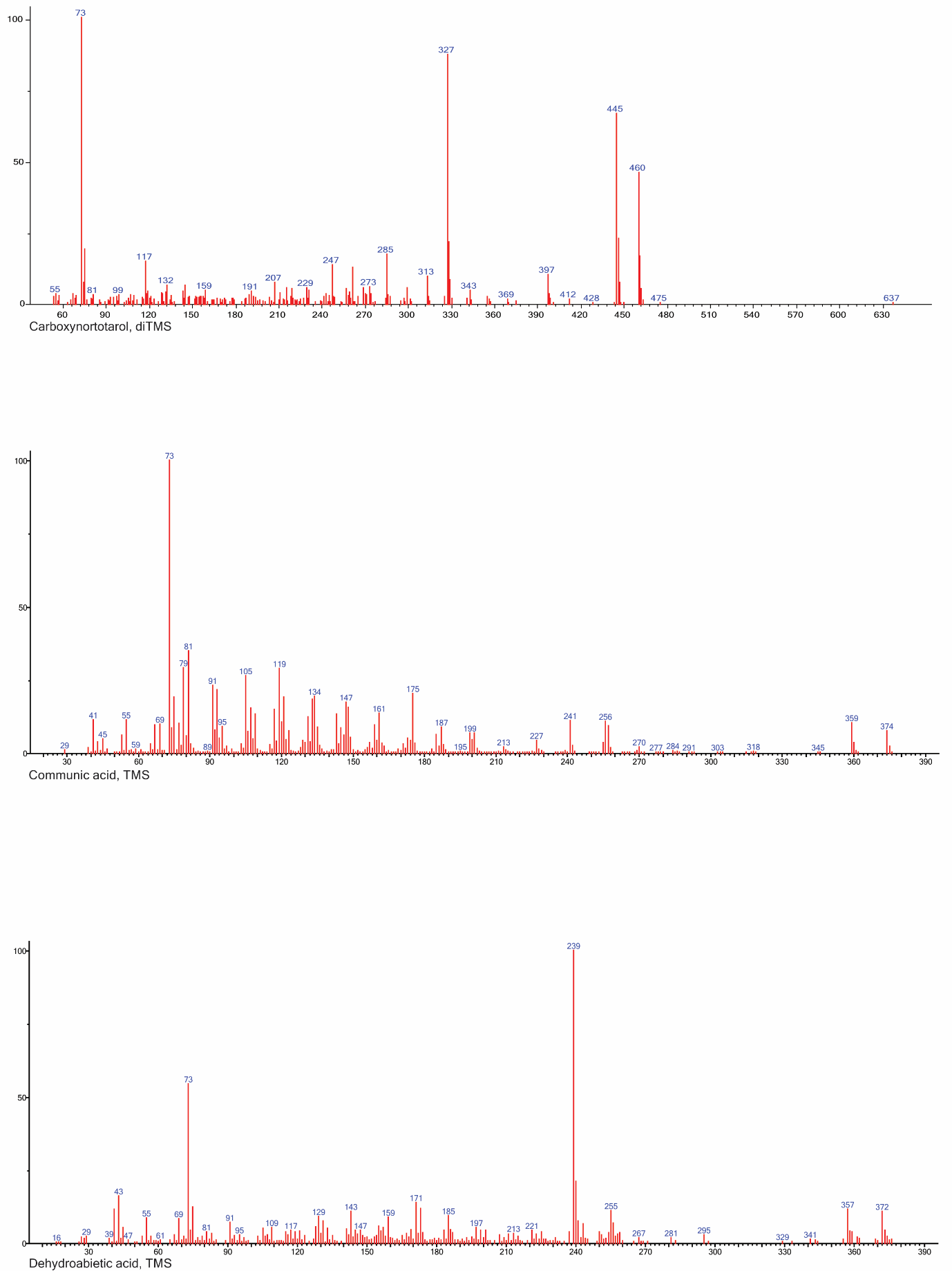


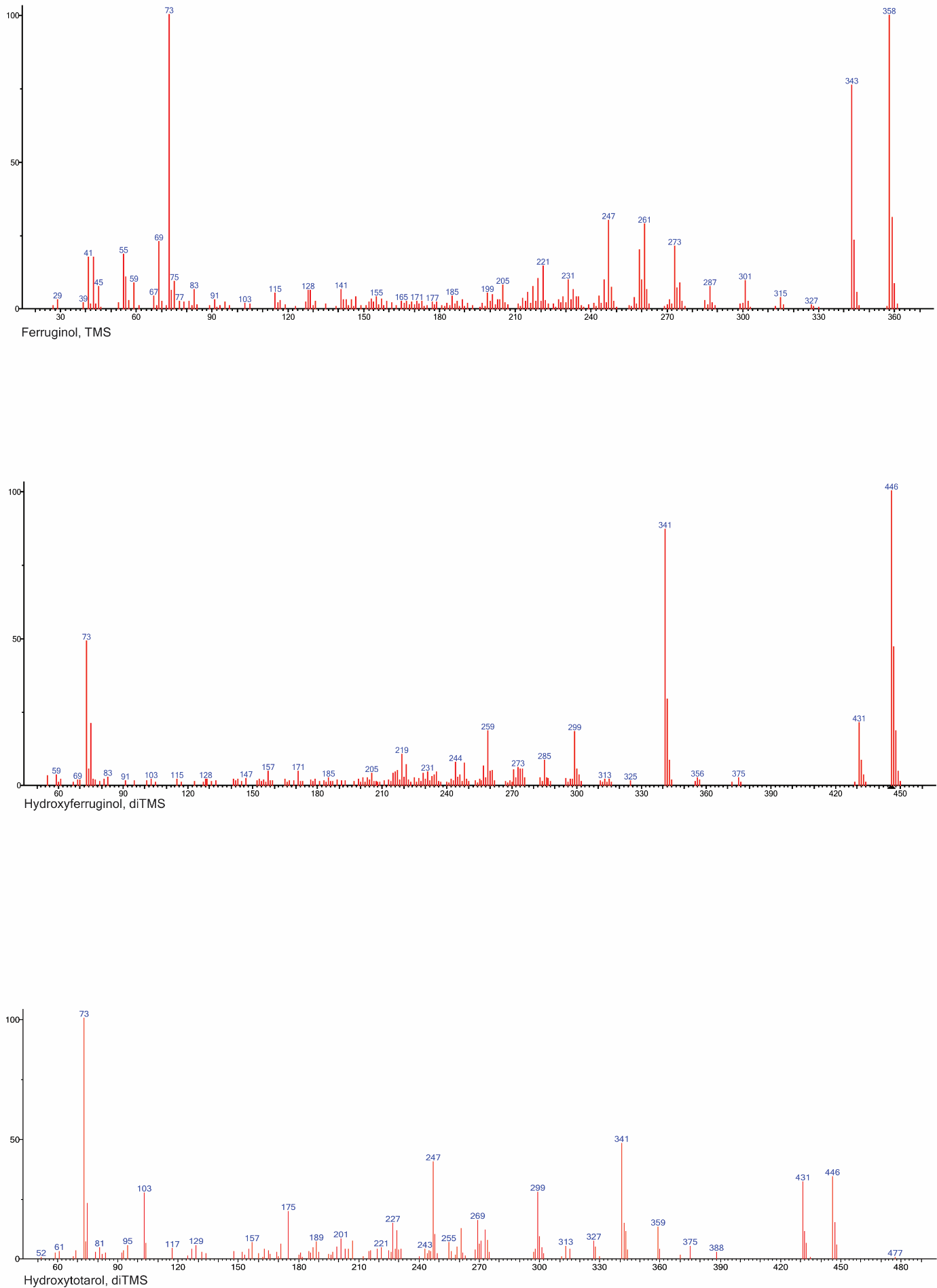


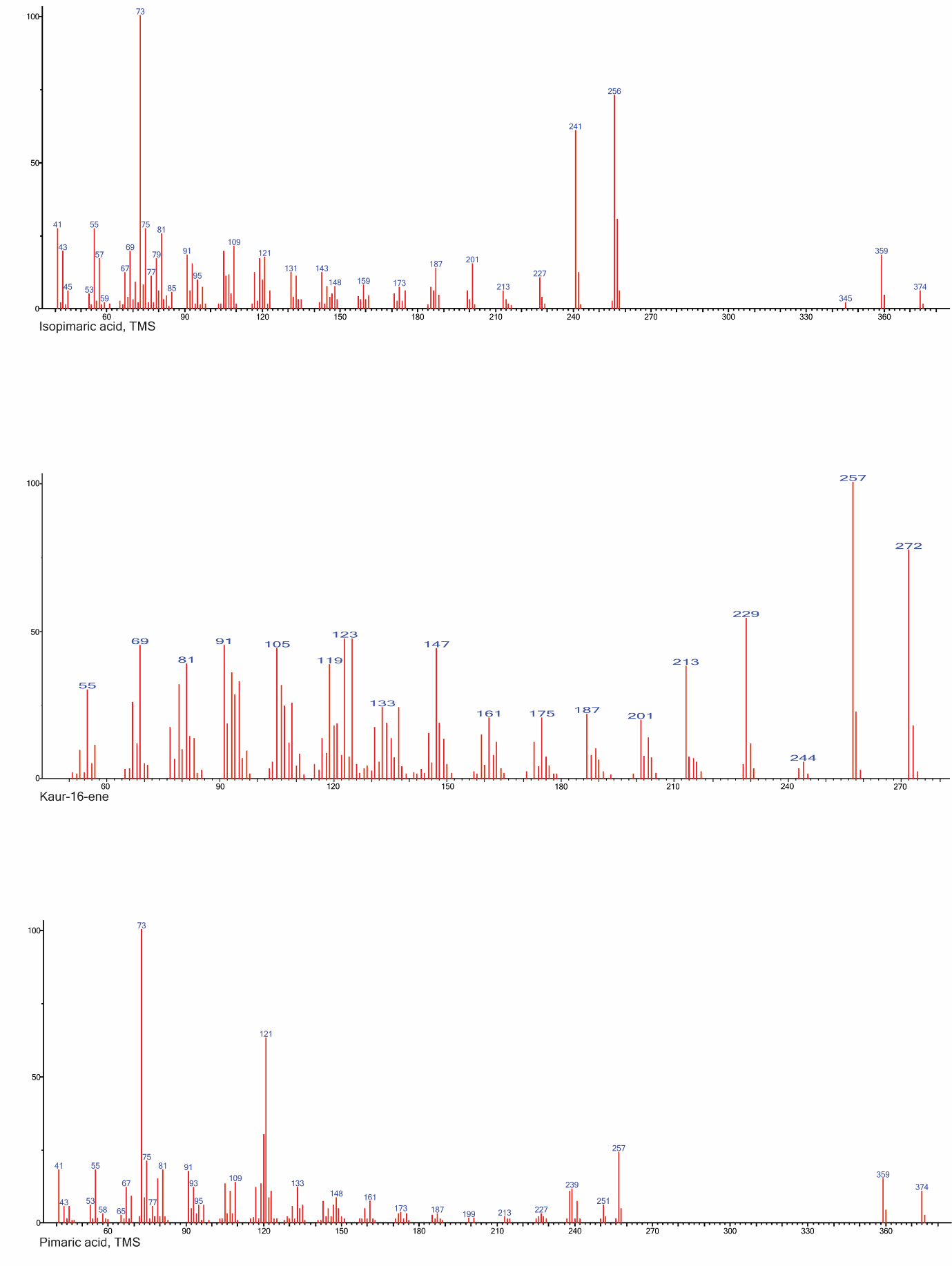


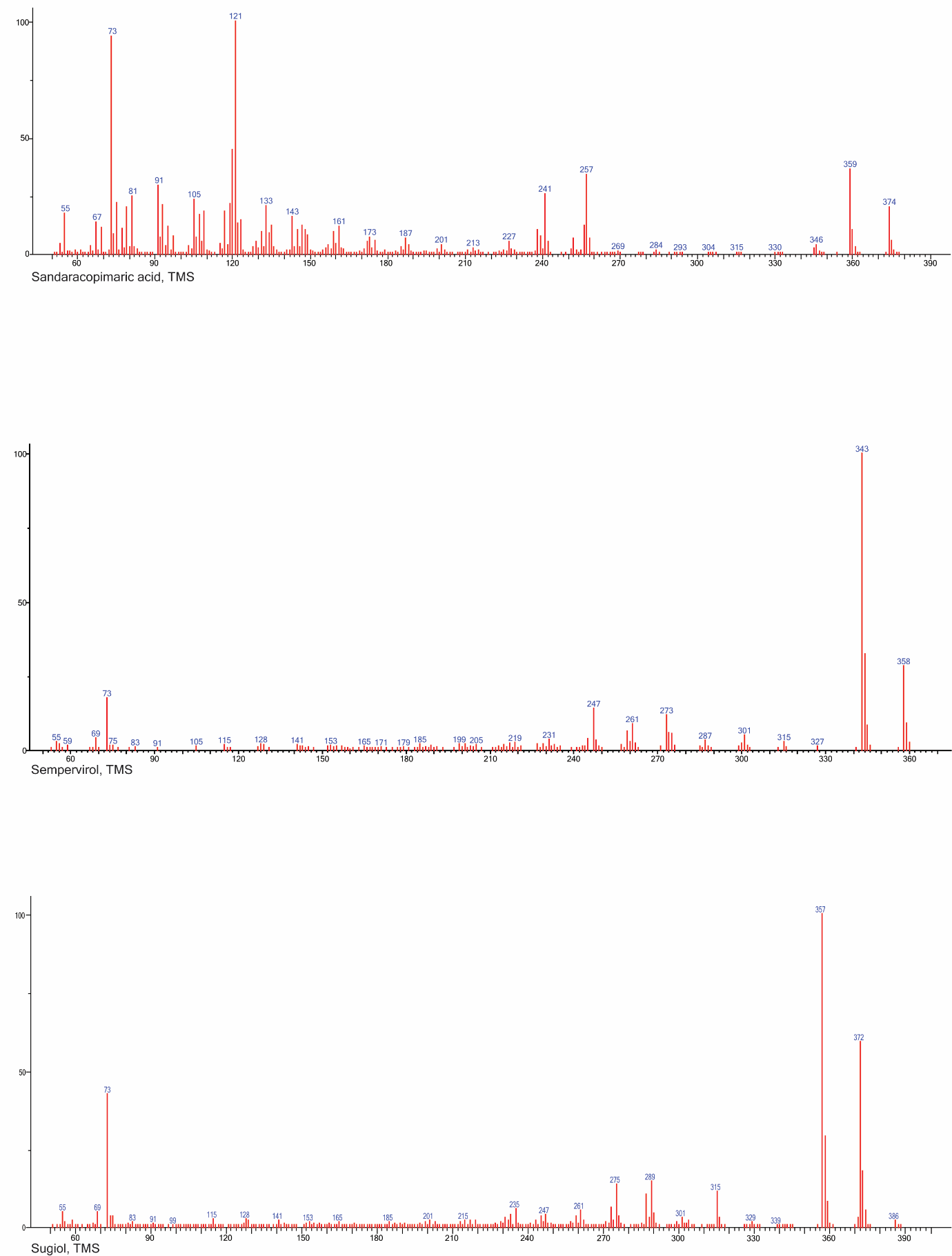


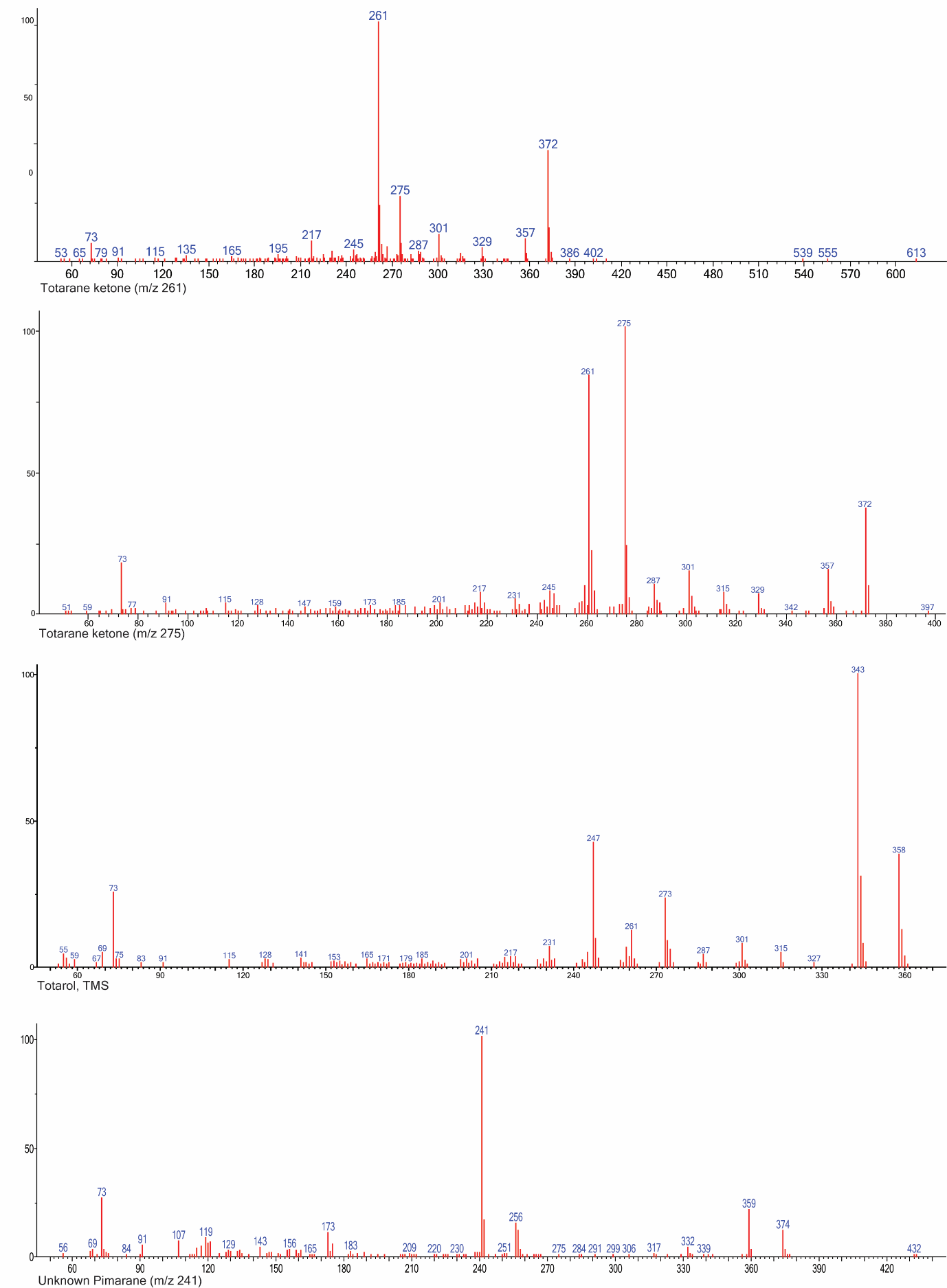

Supplement: S2 File — (DOCX) [file pone.0306402.s002.docx]
